# Effect of Urea on Drug Extraction Efficiency in Reverse Iontophoresis

**DOI:** 10.3390/pharmaceutics17050677

**Published:** 2025-05-21

**Authors:** Rie Yamauchi, Shuji Ohno, Yasuko Obata

**Affiliations:** 1 Division of Pharmaceutical Education and Research, Hoshi University, 2-4-41 Ebara, Shinagawa-ku, Tokyo 142-8501, Japan; ohno@hoshi.ac.jp; 2 Laboratory of Pharmaceutical Science and Technology, Hoshi University, 2-4-41 Ebara, Shinagawa-ku, Tokyo 142-8501, Japan

**Keywords:** reverse iontophoresis, non-invasive drug monitoring, acetaminophen, urea, extraction enhancer, skin hydration, electroosmotic flow

## Abstract

**Background/Objectives**: Reverse iontophoresis (R-IP) is a technology that transdermally delivers components from inside the body to outside the body using electroosmotic flow (EOF) generated by applying a low electric current through the skin. It has attracted attention as a non-invasive sampling method for therapeutic drug monitoring (TDM). The purpose of this study was to determine whether urea and Tween 80 effectively enhance drug extraction from beneath the skin using R-IP. **Methods**: An in vitro drug extraction test using hairless mouse skin and R-IP was performed with a 3-chamber Franz cell and Ag|AgCl electrodes by applying a constant current (0.25 mA/cm^2^) for 6 h. Acetaminophen was chosen as the model drug, and its solution (30, 100, or 300 μg/mL) was placed in the subdermal compartment. The pH of both the electrode and subdermal compartment solutions was maintained at 7.4. **Results**: Acetaminophen was gradually extracted into the electrode compartment in a concentration-dependent manner and was more abundant in the cathode compartment than in the anode compartment. In addition, urea significantly promoted drug extraction, particularly on the cathode side, and a linear relationship was observed between the subdermal concentration and extracted amount. This effect is likely due to skin hydration caused by urea, which enhances EOF generation in the skin. Conversely, Tween 80 had no effect on drug extraction. **Conclusions**: R-IP combined with urea is expected to not only shorten the treatment time but also enable its application to drugs with low concentrations in blood.

## 1. Introduction

Therapeutic drug monitoring (TDM), which was introduced in the early 1970s, is the process of monitoring and measuring drug concentrations in the blood after drug administration, with the aim of providing optimal drug treatment to patients, thus enabling the administration of appropriate drug doses and preventing side effects [1]. However, TDM imposes a burden on patients because blood collection requires puncturing the skin with a syringe needle.

In recent years, a technology called reverse iontophoresis (R-IP) has attracted attention as it bypasses this disadvantage, and fundamental research on its use is underway. R-IP enables transdermal drug transport using electroosmotic flow (EOF), which is generated when a weak electric current derived from iontophoresis (IP) is applied to the skin [1,2]. According to the Nernst–Planck permeation rate equation, the permeation rate of a drug through the skin under an electric field (*J*) is determined by the permeation rate owing to passive diffusion (*J*_p_), electrophoresis (*J*_e_), and EOF (*J*_c_) as follows:

*J* = *J*_p_ + *J*_e_ + *J*_c_ (1)

where *J*_p_ is the flux due to passive delivery calculated from the formula

(2) $$J_{p}=K_{s}D_{s}\;\frac{dC}{h_{s}}$$

*J*_e_ is the flux due to electric current facilitation calculated from the formula

(3) $$J_{e}=\;\frac{Z_{i}D_{i}F}{h_{s}}C_{i}\;\frac{dE}{h_{s}}$$

*J*_c_ is the flux due to convective transport (electroosmosis) calculated from the formula

*J*_c_ = *kC*_s_*I*_d_ (4)

where *K*_s_ is the partition coefficient between the donor solution and the stratum corneum, *C*_i_ is the donor concentration of ionic species *i*, *C*_s_ is the concentration in the skin tissue, d*C*/*h*_s_ is the concentration gradient across the skin, d*E*/*h*_s_ is the electric potential gradient across the skin, *D*_s_ is the diffusivity across the skin, *D*_i_ is the diffusivity of ionic species *i* in the skin, *I*_d_ is the current density, *Z*_i_ is the electric valence of ionic species *i*, *k* is the proportionality constant, and *F* is the Faraday constant.

Ionic drugs are delivered primarily via electrophoresis in a current-dependent manner, whereas electrically neutral molecular drugs are delivered primarily using EOF in a concentration-dependent manner [1,2]. Conventional IP uses the principle of electrophoresis to promote skin permeation of ionic drugs, which are normally difficult for the skin to absorb, via passive diffusion in a current-dependent manner [3,4,5,6,7,8]. In contrast, R-IP targets not only ionic drugs but also electrically neutral molecular drugs, which are transported via the EOF in a concentration-dependent manner. Therefore, if EOF can be generated from inside the body to the outside, it will be possible to achieve the purpose of TDM, which is to extract internal drugs and estimate their concentrations.

EOF is a phenomenon in which counterions forming an electric double layer move when an electric field is applied to a charged interface, causing liquid to flow due to the electric force [1,2]. The conditions for EOF generation in the skin are pH-dependent. For example, when the pH of the skin surface is higher than its isoelectric point, the skin becomes negatively charged and forms an electric double layer with cations, mainly Na^+^ in the solvent [9]. When an electric current is applied under these conditions, water molecules flow from the anode to the cathode as the cations move, and electrically neutral molecular drugs utilizing this flow are extracted from the subdermal region to the outside of the body on the cathode side [1,2,10,11,12,13,14,15]. However, when the pH is lower than the isoelectric point of the skin, the skin becomes positively charged and forms an electric double layer with anions. When a current is applied, the EOF generated by the movement of anions flows from the cathode to the anode, and the molecular drug is extracted on the anode side.

**Figure 1:**
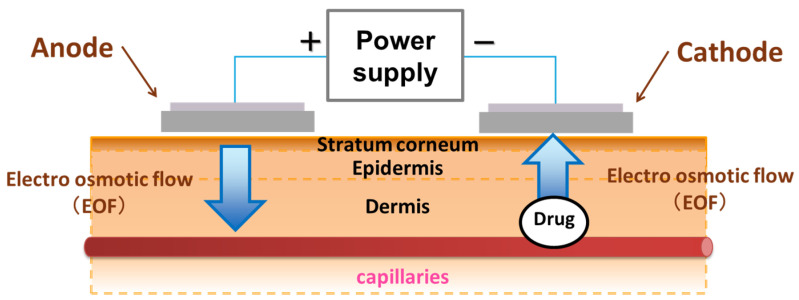
Schematic diagram of in vitro reverse iontophoresis illustrating the electroosmotic flow that occurs from the anode to the cathode when the pH of the electrode solution is higher than the isoelectric point of the skin.

Thus, by adjusting the pH of the skin surface, it is possible to utilize EOF not only to transport drugs from outside the body into the body for transdermal absorption but also to extract drugs from the body to outside the body to monitor drug or endogenous substance concentrations in subcutaneous tissue or blood, as shown in Figure 1.

Furthermore, while conventional IP facilitates current-dependent drug delivery, R-IP utilizes EOF, which enables drug concentration-dependent delivery [1,2]. Therefore, non-invasive sampling during TDM would be possible if EOF can be used to extract drugs to outside the body while reflecting real-time drug concentrations in the body, thus eliminating the need for needle puncture and improving the patients’ quality of life. This technology could be particularly beneficial for individuals who have difficulty undergoing frequent blood collections, such as patients with cancer or AIDS, pregnant women, pediatric and geriatric patients with chronic diseases, and patients who are immobile or unable to report medication side effects due to difficulties in communicating [2].

Examples of basic R-IP research developed in recent years include lithium [16,17], valproic acid [18], salicylic acid [19], nicotine [19], amikacin [20], and gabapentin [21]. It has been reported that drugs can be extracted transdermally via EOF. Many studies have also focused on endogenous substances such as amino acids [22], glucose [12,23], urea [24,25,26], and *l*-lactate [27], with the aim of utilizing them for diagnosing and monitoring metabolic and other diseases. In 2001, GlucoWatch^®^, a medical device that measures blood sugar levels using the R-IP principle, was introduced to the market, but it required calibration and time for drugs to penetrate the skin. Other issues included the need to replace disposable pads every 12 h and device shutdown owing to sweat, ultimately leading to its discontinuation in 2007 [1].

Thus, sufficient knowledge needs to be accumulated to spearhead the development of R-IP technology for clinical application in safe and user-friendly sampling tools that can replace needle-based blood collection for TDM. However, several conditions must be considered for both the drug and the device.

Optimal drug-related conditions [1] include the existence of the drug primarily as an electrically neutral molecule at physiological pH because R-IP relies on EOF rather than electromigration, drugs with high therapeutic concentrations, and drugs with lower protein binding and molecular weights as these properties facilitate tissue permeation, making them advantageous for R-IP. With regard to device-related conditions, appropriate pH must be maintained in the electrode compartment solution upon skin contact to ensure that the EOF occurs in a favorable direction for extraction. Furthermore, the pH should remain close to the physiological conditions to prevent skin irritation. Additionally, buffer solutions and low redox-potential Ag|AgCl electrodes are commonly used to minimize pH fluctuations and gas generation owing to water electrolysis at the area of skin contact [1]. To ensure safety, the device must be capable of stably generating a weak current of 0.5 mA/cm^2^ or less [28,29]. Real-time monitoring is desirable, and the extraction lag time should be minimized. Moreover, the device should offer high extraction reproducibility and be cost-effective. It should also be compact, portable, and ergonomically designed.

Therefore, we deemed it necessary to develop a method for achieving highly accurate and efficient drug extraction within a short period of time, thereby expanding the applicability of R-IP to drugs with low blood concentrations.

**Figure 2:**
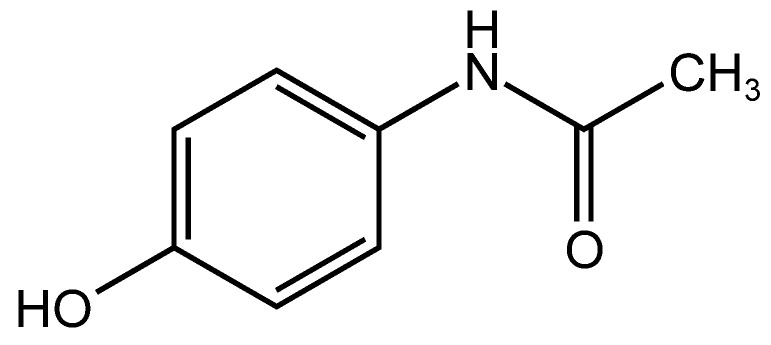
Chemical structure of acetaminophen (M.W., 151.16 g/mol).

In this study, we focused on urea and Tween 80, which have been confirmed as effective and safe transdermal absorption enhancers in IP. We conducted a feasibility study to determine whether they also promote drug extraction via R-IP. As a model drug, we selected acetaminophen (Figure 2), an electrically neutral molecule widely used as a marker of EOF [30,31].

Furthermore, prior to conducting the in vitro transdermal extraction experiments, we designed a new 3-chamber Franz cell. In general, in vitro skin drug permeation tests use either a vertical Franz cell consisting of two chambers or a horizontal two-chamber cell. However, when performing IP or R-IP experiments with these conventional cells, either the anode or cathode is placed in the subdermal compartment, making it impossible to replicate clinical conditions in which both electrodes are positioned on the skin. To address this, we designed a 3-chamber cell based on a previously reported device [21,32]. During the current tests using the newly designed cell, the direction of the EOF was determined based on whether acetaminophen, an electrically neutral drug, was preferentially delivered to the cathode or anode.

## 2. Materials and Methods

### 2.1. Materials

Acetaminophen and Tween 80 were purchased from Tokyo Chemical Industry Co., Ltd. (Tokyo, Japan); methanol and urea were purchased from Nacalai Tesque, Inc. (Kyoto, Japan); ethanol, phosphoric acid, disodium hydrogen phosphate dodecahydrate, sodium dihydrogen phosphate dihydrate, and sodium chloride were purchased from Fujifilm Wako Chemical Corporation (Osaka, Japan). Silver and platinum wires were purchased from Nilaco Corporation (Tokyo, Japan). Labo skin (skin from 7-week-old male hairless mouse) was purchased from Sankyo Labo Service Corporation, Inc. (Tokyo, Japan).

### 2.2. Instruments

A current controller (Potentiostat/Galvanostat HA-151) manufactured by Hokuto Corporation (Tokyo, Japan) was used. A Hitachi Chromaster series system (Hitachi High-Tech Corporation, Tokyo, Japan) consisting of Pump 5160, Auto Sampler 5280, and UV detector 5410 was used to perform high-performance liquid chromatography (HPLC).

### 2.3. Preparation of Electrodes

Ag|AgCl electrodes were prepared by immersing a silver wire and a platinum wire in a 0.9% sodium chloride aqueous solution, connecting them in series, and then applying a constant current of 0.1 mA for 18 h to anodize the silver wire and form a silver chloride coating on its surface.

### 2.4. In Vitro Reverse Iontophoresis (R-IP) Test

**Figure 3:**
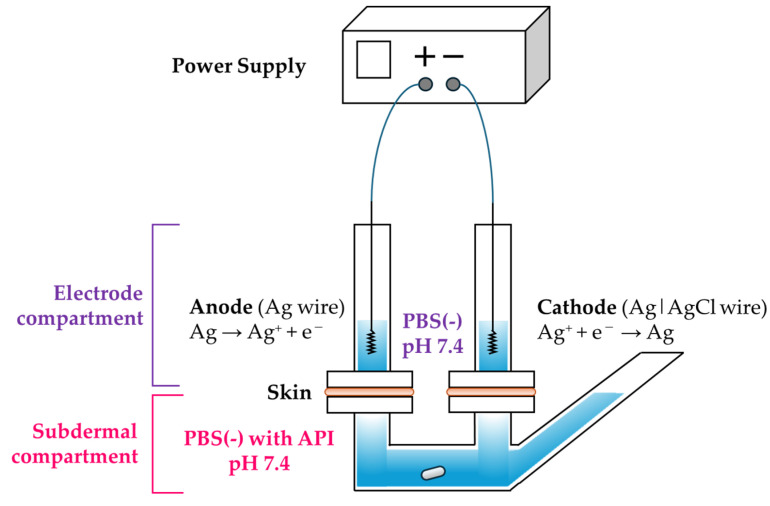
Schematic diagram of the in vitro reverse iontophoretic experimental setup using a 3-chamber Franz cell.

The 3-chamber vertical diffusion cell designed as a 3-chamber Franz cell modified with reference to previous research [21,32] was custom-made by Hario Co., Ltd. (Tokyo, Japan). The experiments were conducted using this 3-chamber Franz cell in which two electrode compartments (internal volume: 4.8 mL) were positioned above the skin and one subdermal compartment (internal volume: 23 mL) was located below the skin (Figure 3). The available surface area of the skin was 1 cm^2^.

Two electrode compartments consisting of an anode and cathode were set on the upper side of the hairless mouse skin, and a subdermal compartment was set on the lower side of the skin. The electrode compartments were filled with Mg^2+^-free phosphate-buffered saline (PBS (−), pH 7.4), PBS (−) containing 1.5% *w*/*v* Tween 80 (pH 7.4), or PBS (−) containing 20% *w*/*v* urea (pH 7.4). For the subdermal compartment, 34 mL of a solution containing various concentrations (30, 100, or 300 μg/mL) of acetaminophen dissolved in PBS (−) (pH 7.4) was injected.

A Ag electrode was connected to the anode of the constant-current generator, whereas a Ag|AgCl electrode was connected to the cathode, with the tip of each electrode placed within the electrode phase. R-IP was performed with a constant current (0.25 mA/cm^2^), and the temperature of the 3-chamber Franz cell was maintained at 32 °C using a water bath. After initiating the in vitro test, 0.5 mL of the electrode phase solution was collected at 1, 2, 3, 4, and 6 h, and the electrode phase was replenished with the same amount of fresh PBS (−) (pH 7.4) as the sampled volume. The collected solution was centrifuged (15,000 rpm for 5 min at 4 °C), and the acetaminophen concentration in the supernatant solution was determined using HPLC.

### 2.5. Determination of Acetaminophen Concentration Using HPLC

The samples were analyzed using reverse-phase HPLC. The concentration of acetaminophen in the sample solution was measured under the following conditions: The HPLC column used was a COSMOSIL 5C18-MS-II column (4.6 mm ID × 150 mm, Nacalai Tesque, Inc. (Kyoto, Japan)). The column temperature was maintained at room temperature, and the detection wavelength was 243 nm. The flow rate was 1.0 mL/min, while the injection volume was 10 µL. The procedure for gradient elusion is described in Table 1. The peak retention time of acetaminophen was 3.3 min. Standard curves were linear over the range of 0.01–10 µg/mL (r^2^ > 0.999), and the minimum limit of quantification (LOQ) was 0.01 µg/mL.

### 2.6. Data Calculation and Analysis

The cumulative amount of acetaminophen extracted per unit area *Q* (µg/cm^2^) can be calculated using the following equation in which *V* is the total volume of the electrode compartment (1 mL), *V*′ is the sampling volume (0.5 mL), and *C_n_* and *C_i_* are the concentrations of acetaminophen (µg/mL) at the *n*-th and *i*-th sampling points, respectively. *A* represents the effective extraction area (cm^2^).

(5) $$Q(µg/{cm}^{2})=(VC_{n}+V^{\prime }\sum_{i=1}^{n-1}C_{i})/A$$

All values are expressed as the mean ± standard deviation (S.D.). The cumulative amount of acetaminophen extracted was plotted against time, and the flux was calculated from the slope of the straight portion of the curve. Linear regression analysis was performed to examine the correlation between the cumulative amount of drug extracted and drug concentration in the subdermal compartment. The effects of chemical enhancers were analyzed using Student’s *t*-test. A *p*-value < 0.05 was considered significant.

## 3. Results

### 3.1. In Vitro Reverse Iontophoresis of Acetaminophen

**Figure 4:**
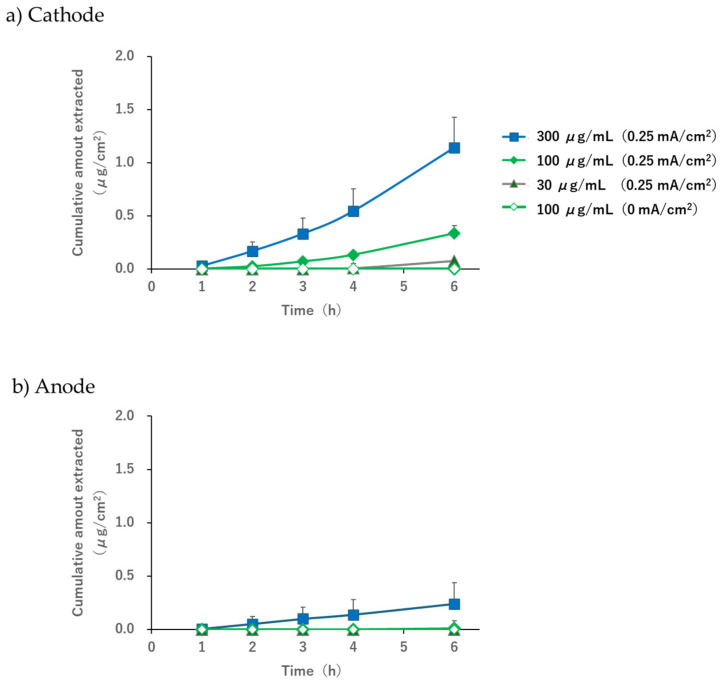
Reverse iontophoresis profiles as a function of time. (**a**) Cumulative amount of acetaminophen extracted in the cathode and (**b**) anode compartments. Each point with a bar represents the mean ± S.D (*n* = 6).

After applying a current of 0.25 mA/cm^2^, acetaminophen was extracted for 6 h (Figure 4). At subdermal concentrations of 30, 100, and 300 μg/mL, the 6 h extraction amounts of acetaminophen with the cathode were 0.08 ± 0.02, 0.34 ± 0.07, and 1.14 ± 0.29 μg/cm^2^, respectively. The 6 h extraction amounts using the anode were 0.01 ± 0.01 and 0.24 ± 0.20 μg/cm^2^ at subdermal concentrations of 100 and 300 μg/mL, respectively, but it was below the detection limit when the subdermal concentration was 30 μg/mL. As mentioned previously, the cathode resulted in a higher extraction amount than the anode. The amount extracted under passive conditions, i.e., when no current was applied for 6 h using both the cathode and anode, remained below the detection limit at a subdermal concentration of 100 μg/mL.

**Figure 5:**
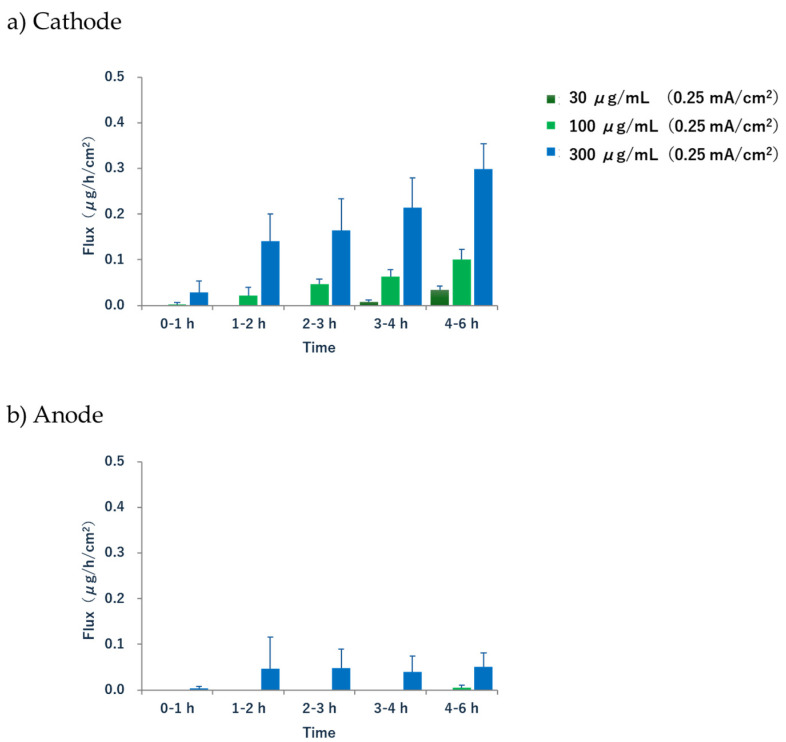
Reverse iontophoresis profiles as a function of time. Extraction flux of acetaminophen in the (**a**) cathode and (**b**) anode compartments. Each point with a bar represents the mean ± S.D (*n* = 6).

The calculation of the extraction rate (flux) at each time point (0–1 h, 1–2 h, 2–3 h, 3–4 h, and 4–6 h) at the cathode showed that the flux continued to increase from 1 h to 6 h after the current was applied (Figure 5). The flux for 4–6 h was 0.03 ± 0.01, 0.10 ± 0.02, and 0.30 ± 0.05 μg/h/cm^2^ at subdermal concentrations of 30, 100, and 300 μg/mL, respectively. In contrast, the anode flux was calculated for a subdermal concentration of 300 μg/mL, and it stopped increasing after 1 h, maintaining a rate of around 0.05 μg/h/cm^2^ for up to 6 h. The extraction rates of acetaminophen at both the cathode and anode were low for up to 1 h but increased significantly from 1 h to 6 h, with the flux never reaching a steady state.

### 3.2. Screening of Chemical Extraction Enhancers

**Figure 6:**
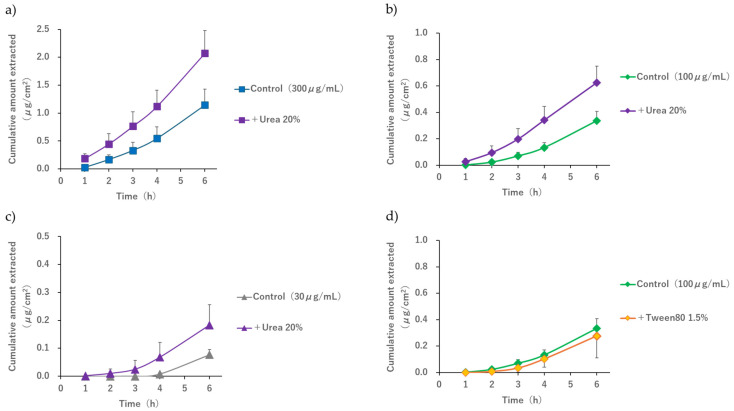
Effect of adding 20% *w*/*v* urea or 1.5% *w*/*v* Tween 80 to the cathodal compartment on the reverse iontophoresis profiles of acetaminophen. Effect of urea on extraction when the subdermal concentrations are (**a**) 300 μg/mL, (**b**) 100 μg/mL, and (**c**) 30 μg/mL. (**d**) Effect of Tween 80 on extraction when the subdermal concentration is 100 μg/mL. Each point with a bar represents the mean ± S.D (*n* = 6).

R-IP was performed at a subdermal concentration of 100 μg/mL to confirm whether adding urea or Tween 80 to the cathodal compartment enhances extraction. When 20% *w*/*v* urea was added to the cathodal compartment solution, significantly enhanced extraction was observed from 1 to 6 h after applying the current (*p* = 0.041 and *p* = 0.021) (Figure 6b). However, when 1.5% *w*/*v* Tween 80 was added, no extraction enhancement was observed (Figure 6d). Furthermore, when we checked whether the addition of 20% *w*/*v* urea to the cathodal compartment enhanced extraction at a subdermal concentration of 300 μg/mL, a significant extraction promoting effect was confirmed at both 1 h and 6 h after applying the current (Figure 6a, *p* = 0.005 and *p* = 0.007). At a subdermal concentration of 30 μg/mL, the extraction amount was too low 1 h after initiating the application of the current to confirm that extraction was enhanced upon adding 20% *w*/*v* urea (*p* = 0.341), but a significant extraction promotion effect was confirmed 6 h later (*p* = 0.010) (Figure 6c).

Table 2 shows the extraction enhancement effect of adding 20% *w*/*v* urea or 1.5% *w*/*v* Tween 80 to PBS (−) as the cathodal solution. When 20% *w*/*v* urea was added, the cumulative extraction amount at 1 h after applying the current was 16 times that of the control. Although the enhancement gradually decreased, a 1.9-fold enhancement was observed even after 6 h. As described above, the extraction enhancement effect due to the addition of urea was noticeable immediately after applying the current, and this effect was maintained throughout the application of the current. Furthermore, when comparing the cumulative extraction amount at the subdermal phase concentrations of 30, 100, and 300 μg/mL with the amount obtained at 6 h in the control (0.08 ± 0.02, 0.34 ± 0.07, and 1.14 ± 0.29 µg/cm^2^, respectively) and at 4 h in the group to which 20% *w*/*v* urea was added (0.07 ± 0.05, 0.38 ± 0.05, and 1.12 ± 0.36 µg/cm^2^, respectively), no significant difference was observed at all concentrations (*p* = 0.7259, 0.9618, and 0.8997, respectively). Moreover, when comparing the amount obtained at 2 h in the control (0.00 ± 0.00, 0.02 ± 0.02, and 0.17 ± 0.09 μg/cm^2^, respectively) and at 1 h in the group to which 20% *w*/*v* urea was added (0.00 ± 0.00, 0.03 ± 0.03, and 0.19 ± 0.09 μg/cm^2^, respectively), no significant difference was observed at all concentrations (*p* = 0.3409, 0.8276, and 0.7404, respectively). However, when 1.5% *w*/*v* Tween 80 was added, the amount of acetaminophen extracted was consistently lower than that in the control. Although there was no statistically significant difference, it was confirmed that the extraction was inhibited.

**Figure 7:**
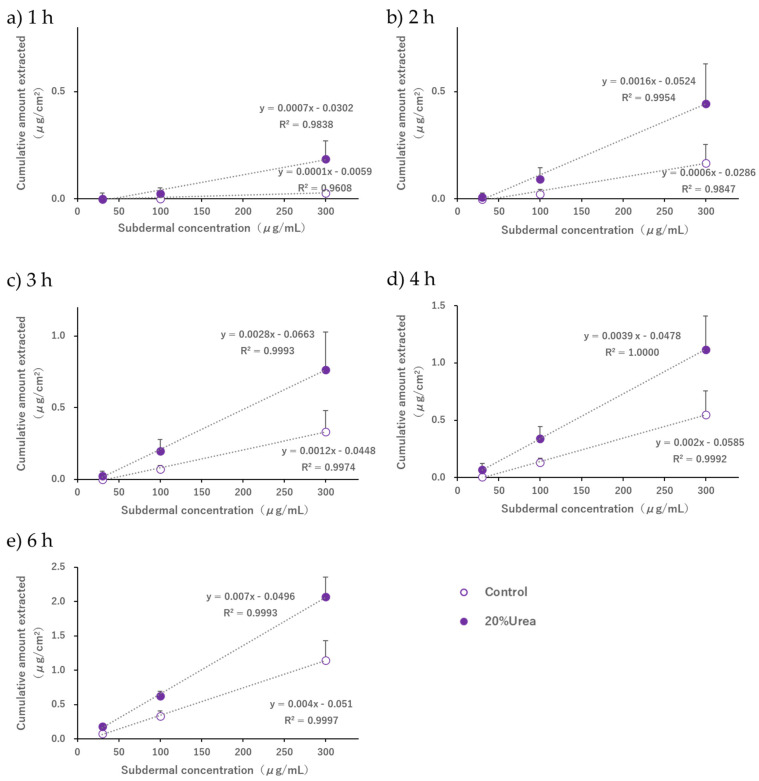
Relationship between the subdermal concentration and the cumulative amount of acetaminophen extracted from the cathodal compartment at (**a**) 1, (**b**) 2, (**c**) 3, (**d**) 4, and (**e**) 6 h with or without the addition of 20% *w*/*v* urea to the cathodal compartment. Each point with a bar represents the mean ± S.D (*n* = 6).

Furthermore, we verified whether the amount of acetaminophen extracted was correlated with its concentration in the subdermal compartment. The extraction amount at the cathode increased linearly with the subdermal concentration from 30 to 300 µg/mL, regardless of whether urea was added or not (Figure 7a–e). The amount of acetaminophen extracted when urea was added increased significantly at every time point from 1 to 6 h after the current was applied, and linearity with the subdermal concentration was confirmed (R^2^ > 0.98).

## 4. Discussion

R-IP is a technique for transdermally delivering drug molecules using EOF generated by passing a low current (approximately 0.5 mA/cm^2^ or less) across the skin. While IP, which relies on electromigration, can deliver only ionic drugs, R-IP can transport electrically neutral polar molecules via EOF. If drugs or endogenous substances present in the blood or tissues can be extracted from the body using the principle of R-IP, this technique can be used in non-invasive sampling tools for TDM. Maximizing the amount of EOF generated in the shortest possible time would effectively facilitate the clinical application of R-IP in TDM.

Therefore, in this study, we focused on urea and Tween 80, which have been reported to enhance the skin permeation of ionic drugs combined with IP, and aimed to investigate whether these substances also promote the extraction of acetaminophen through R-IP.

First, we selected acetaminophen, which is used as a marker for EOF [30,31], as the model drug and performed an R-IP extraction test using a 3-chamber Franz cell. The concentration of acetaminophen in the subdermal compartment was determined based on its kinetic data. The effective blood concentration range of acetaminophen is unclear, but 10 μg/mL acetaminophen has been reported to have antipyretic and analgesic effects [33]. In addition, active metabolites are known to contribute to toxicity, such as liver damage, and if the blood concentration of acetaminophen reaches 200 μg/mL or higher 4 h after ingestion, or 50 μg/mL or higher at 12 h, liver damage is indicated [34]. To encompass these concentration ranges, the concentration of acetaminophen in the subdermal compartment was set at 10 to 300 μg/mL.

When the pH of the electrode phase solution was 7.4, the acetaminophen solution (30, 100, or 300 µg/mL) was placed in the subdermal compartment, and a current of 0.25 mA/cm^2^ was applied; concentration-dependent extraction was observed in the subdermal compartment (Figure 4). Furthermore, the extracted amount and flux at the cathode were higher than those at the anode. It is known that EOF occurs in the anode-to-cathode direction when the pH exceeds the isoelectric point of the skin [1,2,3]. In this study, we used hairless mouse skin, which is reported to have an isoelectric point of approximately 4.5 [35], and performed R-IP in a 3-chamber cell while maintaining the pH at 7.4 with a phosphate buffer. The results of this study reproduced the trends observed in previous studies and confirmed that the conditions required for EOF to occur in the anode-to-cathode direction were successfully replicated.

Some extraction was observed on the anode side only when the subdermal concentration was 300 μg/mL (Figure 4b). Although this study was conducted under conditions in which EOF occurred from the anode to the cathode, it is possible that acetaminophen diffusion followed the concentration gradient slightly. Considering that the extraction amount at a subdermal concentration of 100 μg/mL was below the detection limit for both the cathode and the anode (Figure 4a,b), we concluded that the contribution of passive diffusion was minimal compared to the extracted amount by EOF and was not sufficient to hinder the use of R-IP as a sampling tool for TDM.

The extraction rate (flux) of acetaminophen in the cathode increased significantly after 1–2 h, suggesting a lag between the onset of current application and the initiation of sufficient extraction (Figure 5). In our experimental system using a 3-chamber cell, the drug was initially localized in the solution of the subdermal compartment. This can be considered a multicompartment model, where the drug is delivered to the skin via EOF, with the following order of diffusion and distribution: the subcutaneous compartment → subcutaneous tissue → the epidermis → the stratum corneum. Therefore, there was a lag before significant drug extraction began. Although this lag time may not necessarily be reproduced under in vivo conditions, where the drug may already be present in the subcutaneous tissue immediately after energization, it suggests a possible delay before sufficient EOF, the driving force of R-IP, is generated. Additionally, the extraction rate did not reach a steady state during the 6 h treatment. A similar phenomenon was observed in a previous report on the R-IP of phenytoin, which stated that reaching a steady state is not a clinically essential condition [32]. Our results also showed that the subdermal concentration and the amount of drug extracted were proportional at all time points, and it was possible to estimate the blood concentration by fixing the time from the start of electrical current application to the collection of the extracted sample. Therefore, we consider that the attainment of a steady-state flux is of limited relevance in the context of clinical applications.

Guy et al. [19] extracted drugs noninvasively using R-IP after salicylic acid and nicotine were topically administered (passive loading) to living skin. A kinetic analysis that considered the drug distribution in the subcutaneous tissue compartment and the stratum corneum was performed, and a kinetic model was constructed to explain the process leading to the extraction of intradermal drugs. In this case, the relationship between the amount of drug in the stratum corneum or the living tissue compartment and the rate of drug extraction was approximated using the amount of drug in the adjacent compartments and a first-order rate equation, respectively. Although the conditions of the multicompartment system, which targeted the skin after topical administration, were different from those of our in vitro system, we believe that adopting the linear model concept, in which the concentration of each compartment is proportional to the concentration of the adjacent compartment, would validate the phenomenon in which the amount of drug extracted is linearly related to the concentration in the subdermal compartment, as shown in Figure 7.

When 20% *w*/*v* urea was added during R-IP under conditions where the pH of the electrode phase solution was 7.4 and the acetaminophen concentration in the subdermal compartment ranged from 30 to 300 μg/mL, an extraction enhancement effect was observed up to 6 h after applying the current compared to the control (Figure 6). When comparing the 6 h value of the control group with the 4 h value of the group to which 20% *w*/*v* urea was added or when comparing the 2 h value of the control group with the 1 h value of the group with 20% *w*/*v* urea, no significant difference was observed at any of the subdermal concentrations. This indicated that the 2 h and 6 h values of the group without urea were equivalent to the 1 h and 4 h values of the group with urea, respectively. The above results suggest that, although the extraction amount in 1 to 2 h is small when the subdermal concentration is 30 μg/mL and this condition should strictly be excluded from the discussion, when the concentration is between 100 and 300 μg/mL, performing R-IP while applying urea to the skin surface of hairless mouse may shorten the time required for acetaminophen extraction by 1 to 2 h.

It is well known that the hydration of the stratum corneum, a phenomenon where water loosens the compact packing structure of the stratum corneum, facilitates the skin permeation of many substances [36]. Furthermore, the skin permeation of monoammonium glycyrrhizinate and insulin is enhanced by the combined use of IP and urea [8,37]. We hypothesized that the extraction-enhancing effect of urea confirmed in this study may be due to the hydration of the stratum corneum by urea, which facilitates the formation of aqueous permeation pathways, thereby increasing the EOF. Because the extraction-enhancing effect of urea was noticeable immediately after the start of energization, as shown in Table 2, we considered that the amount of EOF generated—the driving force for drug delivery between multiple compartments in the skin—may have increased immediately after initiating current application because urea promotes the hydration of the stratum corneum. Since the main purpose of this study was to search for candidate compounds that promote drug extraction by R-IP, we have not clarified the mechanism of how urea is involved in enhancing EOF. We would like to clarify in detail the mechanism by which urea affects EOF by investigating, for example, what changes occur in the water content and impedance of the skin when R-IP is performed while applying urea to the skin.

Surfactants, which are used as emulsifiers in various formulations, have also been reported to act as transdermal absorption enhancers [38] and are commonly found in many dermatological formulations. Tween 80, which we focused on as a candidate extraction enhancer, is a nonionic surfactant that promotes the passive diffusion of drugs such as diazepam and lorazepam by disrupting the lipid structure of the stratum corneum [39,40]. Yamamoto et al. reported that the flux of monoammonium glycyrrhizinate, an anionic drug, during the anodal IP of porcine skin was enhanced when combined with either urea or Tween 80 [8]. In this study, although a 1.5% *w*/*v* Tween 80 solution, equivalent to a concentration above the critical micelle concentration (approximately 100 mM) [41], was applied to the skin, no extraction-enhancing effect of Tween 80 was observed for the delivery of acetaminophen using R-IP. Surfactants generally have the potential to solubilize lipids in the stratum corneum [42]. Monomeric surfactants diffuse to the skin surface and act as enhancers by disrupting the lipid structure of the stratum corneum, thereby facilitating diffusion through the barrier phase [43]. The aforementioned effects of the nonionic surfactant Tween 80 on the skin may not improve the rate of material transport when using EOF as the driving force. Furthermore, according to previous reports, when propylene glycol is combined with the IP of acetaminophen, the flux in percutaneous absorption decreases, suggesting that this phenomenon may be due to a decrease in EOF caused by reduced dielectric constant and increased viscosity of the medium [31]. Therefore, because some substances used as chemical enhancers for percutaneous absorption may interfere with EOF-based transport, it is necessary to pay attention to the composition of the solution in contact with the skin to realize the clinical application of R-IP.

This study revealed that adding urea to the electrode phase shortens the time required for drug extraction using R-IP, which not only improves the accuracy of real-time monitoring but also increases the potential for applying R-IP to drugs with low blood concentrations. Furthermore, the shortened drug extraction time contributed to the improved safety of R-IP in clinical applications. This is because there is a nonzero possibility that applying an electric current to the skin may cause skin irritation, and even if the current density is below 0.5 mA/cm^2^, which is considered relatively safe, it is better to apply the electric current for a shorter duration. A slight tingling or itching sensation generally accompanies all IP procedures, and temporary erythema and local vasodilation can occur with the use of IP [3]. Minor skin burns have also been reported [3]. Therefore, shortening the duration of electrical stimulation is a top priority for safe clinical applications.

Urea is widely used in dermatology to improve the skin barrier function and is one of the most common moisturizers and keratolytic agents. Low concentrations (2–10%) of urea are indicated for moisturizing and optimizing the skin’s barrier function, medium concentrations (10–30%) for moisturizers and keratolytic agents, and high concentrations (≥30%) for keratolytic and debriding necrotic tissue. The possibility of increased skin irritation caused by the combined use of R-IP and urea has not been confirmed [44]; however, urea itself causes only moderate skin irritation at high concentrations and is temporary, making it a useful candidate as an extraction enhancer in R-IP.

In this study, we performed R-IP using hairless mouse skin and found that the combination of urea and EOF could effectively promote drug extraction. Although this finding is not conclusive, it may contribute greatly to the clinical application of R-IP. The hairless mouse skin we used in our in vitro test had a thinner stratum corneum, epidermis, and dermis than human skin, which results in conditions of higher drug permeability than when R-IP is performed in human skin, which is a limitation of this study.

Therefore, it is necessary to conduct additional verifications using human or porcine skin, which is histologically similar to human skin [45,46]. Furthermore, to realize the clinical application of R-IP in the future, it is desirable to verify the in vitro–in vivo correlation (IVIVC) to clarify whether the amount of R-IP extracted at any point in time reflects its concentration in body tissues or blood. Given the example of Glucowatch^®^, where the estimated blood glucose level corresponded to the actual blood concentration, albeit with a lag time of 18 min [12,23], it is reasonable to suggest that IVIVC may be established for various drugs delivered using R-IP.

## 5. Conclusions

This study suggests that adding urea to the electrode phase of the R-IP system to promote the hydration of the skin surface may enhance the drug extraction effect of R-IP. Thus, the accuracy of real-time monitoring can be improved by shortening the drug extraction lag. Consequently, R-IP can possibly be applied as a TDM sampling tool under the presence of urea, even for drugs with low blood concentrations, contributing to safety during application.

## Figures and Tables

**Table 1:** Gradient elution procedure for acetaminophen chromatographic conditions.

Time (min)	Flow Rate (mL/min)	A%	B%	C%
0–4	1.0	25	1	74
4–8	1.0	99	1	0
8–12	1.0	25	1	74

A, B, and C were methanol, 10% phosphoric acid, and distilled water, respectively, while A%, B%, and C% are the percentages of mobile phases A, B, and C.

**Table 2:** The enhancement ratio (ER) relative to the control.

Enhancement Ratio	Formulation of the Electrode Compartment		
	PBS (−) (Control)	20% *w*/*v* Urea /PBS (−)	1.5% *w*/*v* Tween 80 /PBS (−)
ER_1 h_	1.0	16.0	ND ^1^
ER_2 h_	1.0	4.1	0.4
ER_3 h_	1.0	2.8	0.5
ER_4 h_	1.0	2.6	0.8
ER_6 h_	1.0	1.9	0.8

^1^ ND: not defined because the cumulative amount extracted was below the detection limit.

## Data Availability

All data and materials in this article are available.
